# All-dielectric metasurface circular dichroism waveplate

**DOI:** 10.1038/srep41893

**Published:** 2017-01-31

**Authors:** Jingpei Hu, Xiaonan Zhao, Yu Lin, Aijiao Zhu, Xiaojun Zhu, Peiji Guo, Bing Cao, Chinhua Wang

**Affiliations:** 1 College of Physics, Optoelectronics and Energy & Collaborative Innovation Center of Suzhou Nano Science and Technology, Soochow University, Suzhou 215006, China; 2 Key Lab of Advanced Optical Manufacturing Technologies of Jiangsu Province & Key Lab of Modern Optical Technologies of Education Ministry of China, Soochow University, Suzhou 215006, China; 3 School of Electronics and Information, Nantong University, Nantong 226019, Jiangsu, China

## Abstract

We propose and experimentally demonstrate a high efficient circularly polarizing dichroism waveplate (CPDW) using a Si-based all-dielectric 2Dchiral metasurface. We demonstrate that the CPDW exhibits a unique dichroism in that it functions as a transmissive quarter waveplate for one of either left-or right-handed circularly polarized incident lightand a reflective mirror for the opposite polarization. The circular polarization dichroism (CPD = I_RCP_ − I_LCP_) in transmission at wavelength ~1.5 μm reaches 97% and the extinction ratio (ER = I_RCP_/I_LCP_) is as high as 345:1. Experimental fabrications and measurements of the proposed all-dielectric metasurface are implemented and found to be in excellent agreement with the simulations. The proposed all-dielectric chiral metasurface is of advantages of high-dichroism, easy-fabrication and standard semiconductor fabrication techniques compatible, which could lead to enhanced security in fiber and free-space communications, as well as imaging and sensing applications for circularly polarized light with a highly integrated photonic platform.

Circularly polarizing dichroism (CPD) has attracted much attentions recently due to its wide applications in various optical techniques and devices^1^,^2^,^3^,^4^,^5^,^6^. In circular polarization, the electric field vector travels along a helical trajectory, either clockwise or counterclockwise (i.e., right-handed or left-handed circular polarization)^7^. Circularly polarizing dichroism represents different transmissions (or reflections) of left-handed and right-handed circularly polarized incident waves upon a photonic device.

Over the past few years, advances in surface plasmonic polaritons opened an avenue for designing compact functional circularly polarizing device on subwavelength scale. In 2009, Gansel *et al*. proposed and demonstrated a 3-dimensioanl (3D) helical nano-wire circular polarizer in the IR range^8^, in which a circular polarization dichroism of as high as 80% (CPD = I_RCP_ − I_LCP_ with I_RCP_ and I_LCP_ being the transmission of right-handed and left-handed polarization) was achieved. The physical mechanism of CPD behind the 3D helical nano-wire structure is the phase matching between the polarized electric vector along the propagating direction and surface plasmonics along the spatial spiral direction of the 3D helical nano-wires. Following the concept of the 3D helical wires, different versions of the helical wires based CPD devices were investigated, which include tapered gold-helix metamaterials^9^ multi-cycle double-helical intertwined^10^ or multi-cycle multiple-helices intertwined^11^,^12^,^13^ 3D wire structures. Zhao *et al*. also proposed a stacked nanorod metamaterials arrays with a tailored rotational twist, from which the CPD reaches 70% theoretically, but 30% experimentally due to losses through multilayer metals^14^. These 3D chiral structures exhibit great potential and excellent behavior in circular polarization manipulation, the fabrication and integration of these 3D metal-wire structures is, however, very complicated.

The CPD with planar plasmonic structures was also investigated. Fedotov *et al*. reported that asymmetric transmission of a circularly polarized light through a planar metal nanostructure consisting of continuous chiral “fish-scale” elements can be observed and a maximum CPD of 25% in the visible to near-IR spectral region can be achieved^15^. Two dimensional (2D) Archimedean plasmonic spirals have also shown capabilities for circular polarization analyzing^16^,^17^, in which different transmission patterns with either a bright spot or a donut shape corresponding to left or right circular polarized incidence can be obtained. The energy of the total transmission of left or right circular polarized incidence is, however, indistinguishable. In 2015, Li *et al*. demonstrated a chiral circular dichroism metamaterial consisting of a periodic array of ‘Z’-shaped silver (Ag) chiral metamolecules, from which a CPD of 90% in the reflection mode was achieved due to chiral metal-atom anisotropic absorption^18^.

All-dielectric based nano devices have been attracting much attention because of their low losses in the visible and near-infrared spectral range and compatible with standard semiconductor fabrication techniques. In 2011, Konishi *et al*. designed and fabricated a GaAs-based semiconductor chiral gammadion photonic crystal incorporated with an InAs quantum dot (QD) layer that produces circularly polarized photoluminescence (PL). Due to interaction between the chiral crystal and the photons radiated from the QDs, a strong circular anisotropy from the chiral nanostructure was observed in both simulation and experiment, and a maximum experimental circular polarization degree of 26% was achieved (circular polarization degree = (I_RCP_ − I_LCP_)/(I_RCP_ + I_LCP_), I_LCP_ and I_RCP_ are the emission intensities of the left and right circular polarization)^19^. In 2014, Maksimov *et al*. improved the single layer chiral gammadion structure with multilayer (~20 layers) chiral gammadion structure of different materials, from which a circular polarization degree of 81% in experiment was obtained^20^. Very recently, Lobanov *et al*. further proposed a chiral nanostructure in which gammadions are replaced by four rectangles, each rotated by 90° with respect to its nearest neighbors^21^. A circular polarization degree around 99% of the emission was predicted theoretically and up to 96% was achieved experimentally^22^. It is noted that although the emission from gammadions based structures is of high circular polarization degree, the circular polarization dichroism is still low because of the limited transmission from the structure. Also in 2015, Wu *et al*. proposed a 2D chiral silicon based metasurface for circularly polarized infrared radiation in the wavelength band of 3–5 μm 23. Each unit cell of the structure comprises one straight and one “L-shape” Si nanorods, from which a CPD of 70% can be achieved in a very narrow wavelength region (~20 nm).

In this paper, we propose and experimentally demonstrate a high efficient circularly polarizing dichroism waveplate (CPDW) using an all-dielectric 2D chiral metasurface that is based on CMOS-compatible materials: silicon and its oxide, in which a simple Z-shaped arrays are etched into an ultra-thin silicon layer (~200 nm) on top of a SiO_2_ substrate with a very small aspect ratio (depth/width ~0.7). It is shown that the CPDW exhibits a unique dichroism in that it functions as a transmissive quarter waveplate for one of either left- or right-handed circularly polarized incident light and a reflective mirror for the other. The CPD in the transmission at wavelength ~1.5 um reaches 97% and the extinction ratio is as high as 345:1. Experimental fabrications of the proposed all-dielectric metasurface structure are implemented with electron beam lithography and inductively coupled plasma etching. Experimental demonstrations of the CPDW are consistent with the simulations. The proposed all-dielectric 2D chiral metasurface is of advantages of high-dichroism, easy-fabrication and able to use standard CMOS-compatible semiconductor fabrication techniques, which could provide useful devices for remote sensing and imaging applications for circularly polarized light with a highly integrated photonic platform.

## Results

### Chiral metamaterial and device design

**Figure 1:**
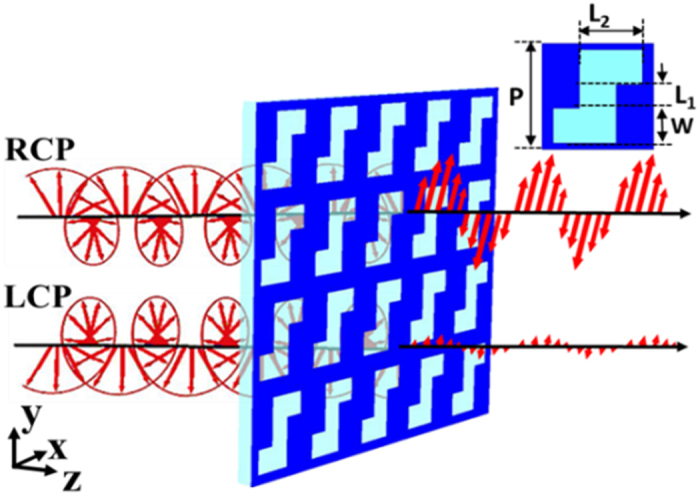
Schematic diagram of a circularly polarizing dichroism waveplate (CPDW).

The proposed chiral metasurface (Fig. 1) is an all-dielectric periodic array with a unit cell consisting of a Z-shaped slot antenna etched into a silicon thin film (film thickness: H = 0.215 μm) that is deposited on a silicon oxide substrate. The inset figure shows the schematic diagram of a Z-shaped unit cell, in which L_1_, L_2_, W and P stand for the perpendicular beam, horizontal beam, width of the slot and the period of the unit cell, respectively.

**Figure 2:**
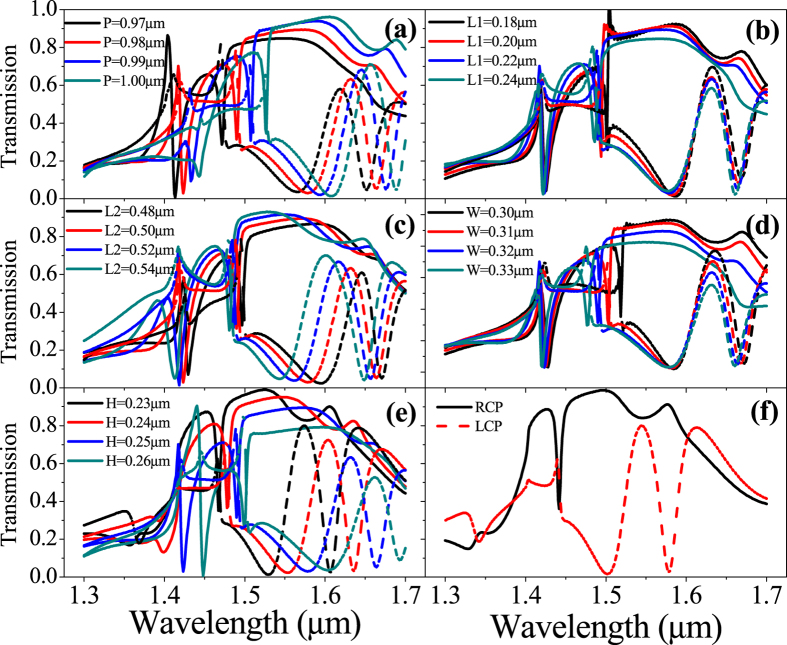
Transmission spectra of different circularly polarized incident lights. Transmission spectra (RCP: solid line and LCP: dashed line) as a function of (**a**) P; (**b**) L_1_; (**c**) L_2_; (**d**) W and (**e**) H. In the simulations, P = 0.98 μm, L_1_ = 0.22 μm, L_2_ = 0.50 μm, W = 0.32 μm and H = 0.25 μm, unless specific indication. (**f**) An optimized transmission spectra with the best extinction ratio: P = 0.98 μm, L_1_ = 0.22 μm, L_2_ = 0.50 μm, W = 0.32 μm and H = 0.215 μm (RCP: black solid line and LCP: red dashed line).

The effects of different parameters of the structure on the performance of different circular polarizations in the wavelength range of 1.3–1.7 μm are firstly investigated. Figure 2(a) shows the effect of the period P on the transmission of LCP and RCP incidences. It is seen that the resonant wavelengths of both LCP and RCP incident polarizations shift sensitively towards longer wavelengths with the increased period P. The RCP incidence passes through the structure with high transmission except two resonant valleys at ~1.40–1.45 μm and ~1.5 μm induced by longitudinal waveguide structure formed by substrate-metasurface (Si)-air along the light propagation direction (Z-axis). In contrast, four resonant valleys appear in the case of LCP incidence (dashed line), in which two valleys are around is ~1.40–1.45 μm and ~1.5 μm (same positions as that in RCP incidence), the other two valleys are at ~1.58 μm and ~1.67 μm, respectively. The first two valleys at ~1.45 μmand 1.5 μm are from the same longitudinal waveguide resonant effect as that of RCP incidence (common waveguide effect for both RCP and LCP), while the third valley at ~1.58 μm and the fourth valley at ~1.67 μm are from the transverse waveguide effect formed by Si ridged core along Y axis cladded by Z-shaped air slots along Y axis (Fig. 1). The transverse waveguide effect is of chiral characteristics (chiral waveguide effect due to in-plane chiral structure) and is, therefore, sensitive to the incident polarizations. The high dichroism in transmission induced by the chiral transverse waveguide to different circular polarizations forms the fundamentals of the proposed CPDW structure. The behaviors shown in Fig. 2(a) suggest that the operating waveband of the structure can be tuned by manipulating the period across the desired wavelength regime. Figure 2(b) shows the effect of the perpendicular beam L_1_ on the transmission of LCP and RCP incidences. It is seen that the valley positions of both RCP and LCP transmission remainalmost unchanged. In contrast to the effect of L_1_, the horizontal beam L_2_ plays an important role on the operating bandwidth of the transmission dichroism. As shown in Fig. 2(c), the transmission valley at ~1.58 μm of LCP incidence shiftssensitively to shorter wavelength as L_2_ increases, while the valleys at 1.4–1.45 μm of LCP incidence and ~1.5 μm of RCP incidence shift very slightly. The phenomena shown in Fig. 2(b) and (c) can be well explained by the longitudinal and the chiral transverse waveguide effects. While changes in L_1_and L_2_ do not change the longitudinal waveguide structure significantly (both the waveguide dimension and effective refractive index of core and cladding formed by substrate-metasurface (Si)-air), i.e., both valleys at 1.4–1.45 μm of LCP incidence and at ~1.5 μm of RCP incidence remain almost unchanged, changes in L_2_ do change the chiral transverse waveguide mode in which the width of chiral Si ridges (i.e., core of the transverse waveguide along Y-axis) and the corresponding width of the effective cladding formed by the Z-slot along Y-axis change as L_2_ changes, which results in a significant dichroism valley shift of LCP incidence as shown in Fig. 2(c). The same phenomena can also be seen in Fig. 2(d) in which changes in Z-slot width (W) do not change both the longitudinal and chiral transverse waveguide effects significantly, which results in insignificant shifts in all the resonant valleys. Figure 2(e) shows the circular polarization dichroism of the structureon the thickness of the silicon film. As expected, all the resonant valleys corresponding to both RCP and LCP incidences are sensitive to the Si thickness, because both the longitudinal and transverse waveguide structures are altered as the thickness of Si-ridge changes. It is seen from Fig. 2(e) that the operating bandwidth and the polarization dichroism can be tuned by the thickness of the Si film in which a trade-off can be obtained between the bandwidth and the dichroism. Figure 2(f) shows an optimized circular polarization dichroism in which a CPD of 97% (I_RCP_ − I_LCP_) and an extinction ratio (I_RCP_/I_LCP_) of 345:1 have been achieved with the structural parameters: P = 0.98 μm, L_1_ = 0.22 μm, L_2_ = 0.50 μm W = 0.32 μm and H = 0.215 μm.

**Figure 3:**
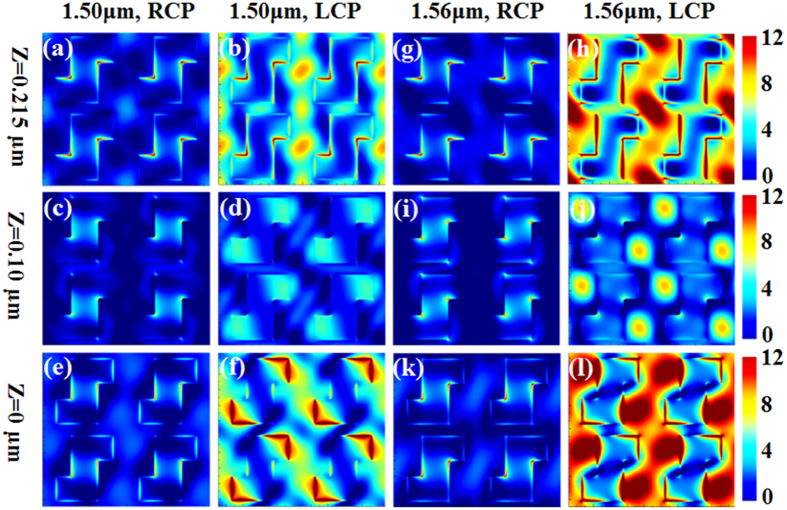
The electric field distribution at different cross-sections of the chiral waveguide layer in four unit cells with different circularly polarized incidences on a left-handed all-dielectric chiral structure. The first row is at air-Si interface (z = 0.215 μm), the second row is in the middle of Si layer (z = 0.10 μm) and the third row is at substrate-Si interface (z = 0). The calculation parameters: P = 0.98 μm, L_1_ = 0.22 μm, L_2_ = 0.50 μm, W = 0.32 μm and H = 0.215 μm.

The “Z-shaped” transverse waveguide is chiral in structure (i.e., the “Z-shaped” slot cannot be superimposed on its mirror image). The chiral structure will result in circular dichroism, i.e., different optical responses (e.g., reflection, transmission or absorption) for left-handed circularly polarized (LCP) incident light and right-handed circularly polarized (RCP) incident light, which has been observed and reported^14^,^18^,^24^. The observed chiral asymmetry in transmission between the left and right circular polarized incident light shown in Fig. 2 can also be demonstrated with the distribution of electromagnetic field within the chiral transverse waveguide metasurface structure. Figure 3 shows the electric field distribution at different cross-sections of the substrate-metasurface (Si)-air waveguide at the chiral resonant wavelengths 1.50 μm and 1.56 μm. Electric field distributions of RCP and LCP incidences are plotted, respectively, at Z = 0 (i.e., interface between SiO_2_ substrate and Si metasurface), Z = 0.10 μm (i.e., middle inside Si metasurface) and Z = 0.215 μm (i.e., top interface between Si metasurface and air). As can be seen, significant differences in the electric field distribution can be seen in the RCP and LCP incidences at both chiral resonant wavelengths of 1.50 μm (the 1^st^ and 2^nd^ column) and 1.56 μm (the 3^rd^ and 4^th^ column). In the case of LCP incidence at both resonant wavelengths (2^nd^ and 4^th^ column), it is seen that very strong light localization happens within the chiral transverse waveguide formed by the Si ridged core along Y axis cladded by Z-shaped air slots along Y axis due to the strong coupling interaction when the handedness of incident circular polarization and the handedness of structure matches. It is also noticed that major part of the energy in the transverse waveguide are near the interfaces at Z = 0 and Z = 0.215 μm. In contrast, in the case of RCP incidence, negligible light localization can be seen in the transverse waveguide (Si-ridge core) at all the different cross sections, which implies that no strong coupling interaction occurs in the case of opposite handedness of the chiral structure and polarized incidence, so that all the RCP incidence transmits through the structure, as witnessed in Fig. 2.

The strong dichroism in transmission of the two circular polarization states through the proposed 2D chiral dielectric metasurface makes it very distinct from 2D chiral metallic metasurfaces that rely on either Ohmic dissipation or symmetry-breaking substrate effects^15^,^25^ to achieve such a transmission asymmetry. Numerical simulations (not shown) indicate that even in the absence of substrate and dissipation (which is negligible in Si for mid-IR frequencies) it is possible for the transmission of the RCP incidence to be different from the transmission of the LCP incidence.

**Figure 4:**
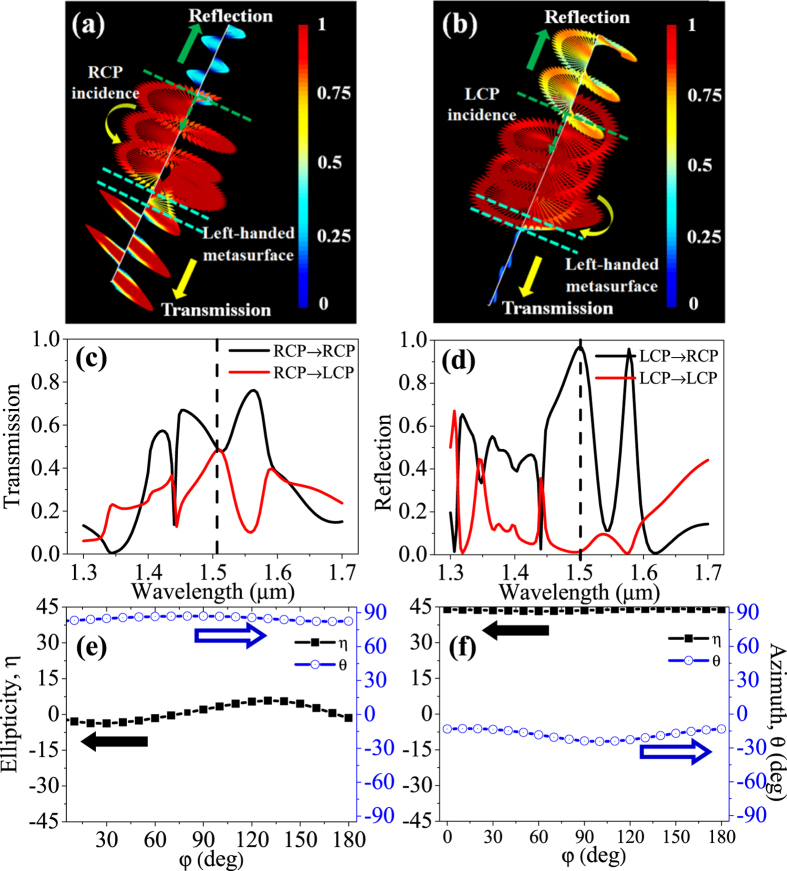
Analysis of polarization state in transmission and reflection from the proposed all-dielectric chiral structure. (**a**) RCP incidence. (**b**) LCP incidence. The amplitude of the transmitted and reflected electrical field is encoded by the color bar. (**c**) RCP and LCP components spectra in transmission when a RCP incident light transmits through a left-handed metasurface. (**d**) RCP and LCP components spectra in reflection when a LCP incident light is reflected from the left-handed metasurface. (**e**) Polarization ellipticity (η) and azimuth angle (θ) of the transmitted light with a linearly polarized incident light at wavelength 1.5 μm; (**f**) Polarization ellipticity (η) and azimuth angle (θ) of the reflected light with a linearly polarized incident light at wavelength 1.5 μm. Parameters are P = 0.98 μm, L_1_ = 0.22 μm, L_2_ = 0.50 μm, W = 0.32 μm and H = 0.215 μm.

The detailed polarization state of the transmission and reflection from the proposed 2D chiral metasurface can be further examined both numerically and analytically. Figure 4(a) and (b) show the numerically simulated polarization state of the transmission and reflection of a RCP and a LCP incidences on the left-handed chiral metasurface at wavelength of 1.50 μm. It is clear that the RCP incident light almost transmits through the all-dielectric left-handed chiral metasurface with very small residual reflection, while the LCP incident light is almost reflected with very small transmission, which are shown in the movies in Supporting information. The detailed quantitative transmission and reflection corresponding to RCP and LCP incidences are also given in Supporting information. Detailed observations on Fig. 4(a) and (b) reveal that the polarization states of the transmission and the reflection experience different changes upon transmitted through or reflected on the 2D chiral surface. It is seen that the transmitted light becomes essentially a linearly polarized light (Fig. 4(a)), i.e., the chiral metasurface functions as a quarter waveplate in transmission, while the reflected light remains circularly polarized (Fig. 4(b)), i.e., the metasurface functions as a mirror, which is a unique function of the proposed structure.

A detailed quantitative analysis regarding the transformation of the polarization state of a RCP and LCP incidences is shown in Fig. 4(c) and (d). Figure 4(c) shows the RCP and LCP components in transmission when a RCP light is incident onto the left-handed chiral metasurface. It is seen that the both RCP and LCP components exist in the transmission when a RCP light is incident onto the left-handed metasurface. The overall polarization state of the transmission is a vector summation of the co-existed RCP and LCP components in the transmission. It is known that a superposition of a RCP and a LCP light with equal intensity generates a linear polarization, and also a linear polarization can be decomposed into a RCP and a LCP light vice versa^26^:


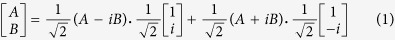


where A and B are the polarization components along X and Y axis, respectively, in Cartesian coordinate system. It is then clear that a linear polarization can be obtained at the wavelength of ~1.50 μm, where the intensity of RCP and LCP components in transmission are equal, which is consistent with that numerically observed in Fig. 4(a). A generalized ellipticity and polarization azimuth angle of the transmitted light can be expressed in terms of RCP and LCP components with ellipticity, η = atan[(I_RCP_ − I_LCP_)/(I_RCP_ + I_LCP_)] and polarization azimuth angle θ = [arg(A_RCP_) − arg(A_LCP_)]/2, where A_RCP_ and A_LCP_ denote the complex transmission coefficients of RCP and LCP waves, and arg represents the phase angle^27^. At the resonant wavelength of 1.50 μm, the ellipticity of the transmitted light with RCP incidence is 0.2 and 85.6°, respectively, compared to η = 0 and θ = 90° of a linear polarization theoretically. Similar to that in the transmission of a RCP incidence, Fig. 4(d) shows the RCP and LCP components in reflection when a LCP light is incident onto the left-handed chiral metasurface. As expected, RCP component dominates in the reflection (I_RCP_ ≈ 1) while LCP component in the reflection is nearly zero (I_LCP_ ≈ 0) at the wavelength of 1.5 μmin the case of a LCP incidence. This results in a circular polarized light in the reflection with ellipticity and azimuth angle being 44.2 and −8.5°, respectively, compared to η = 45 and θ = 0° of a circular polarization theoretically, which is again consistent with that numerically observed in Fig. 4(b). It is also noticed from Fig. 4(c) that both the high RCP and LCP components at wavelength ~1.5 μm contribute to a total high transmission for a RCP incidence, and subsequently a high circular dichroism when compared with other structures^23^.

The unique behavior of the proposed circular dichroism device can be further understood with a linearly polarized incident lightof different azimuth angles (φ, deg). Figure 4(e) and (f) shows the ellipticity and polarization azimuth angle of the transmitted and reflected light, respectively, when a linearly polarized light at wavelength of 1.5 μm with different polarization azimuth angles (with respect to X-axis) is incident onto the designed chiral metasurface. As seen in Eq. (1), a linear polarization can be decomposed into a RCP and LCP components, from which different polarization states can be obtained in the transmission and the reflection, respectively, based on the unique function discussed above. It is seen from Fig. 4(e) that a linearlyor near-linearly polarized light is obtained (originates from the RCP component after decomposition of a linear incident polarization, as seen in Fig. 4(a)) in the transmission with an ellipticity close to zero and a fixed azimuth angle (θ) at ~85° no matter what the polarization azimuth angle of the incident light is, which means that the transmitted light is always a linearly or near-linearly polarized light with invariable azimuth angle that is independent of the incident light. Similarly, in Fig. 4(f), it is seen that a circularly polarized light can be obtained in the reflection from a linearly polarized incidence (originates from the LCP component of the linearly polarized incidence, as seen in Fig. 4(b)), in which the ellipticity is also a constant at 44 and the polarization azimuth angle is almost fixed. This means that the reflected light of a linearly polarized incidence is always an elliptically polarized light with a high ellipticity (very close to a circular polarization).

### Fabrication and measurements of the device

**Figure 5:**
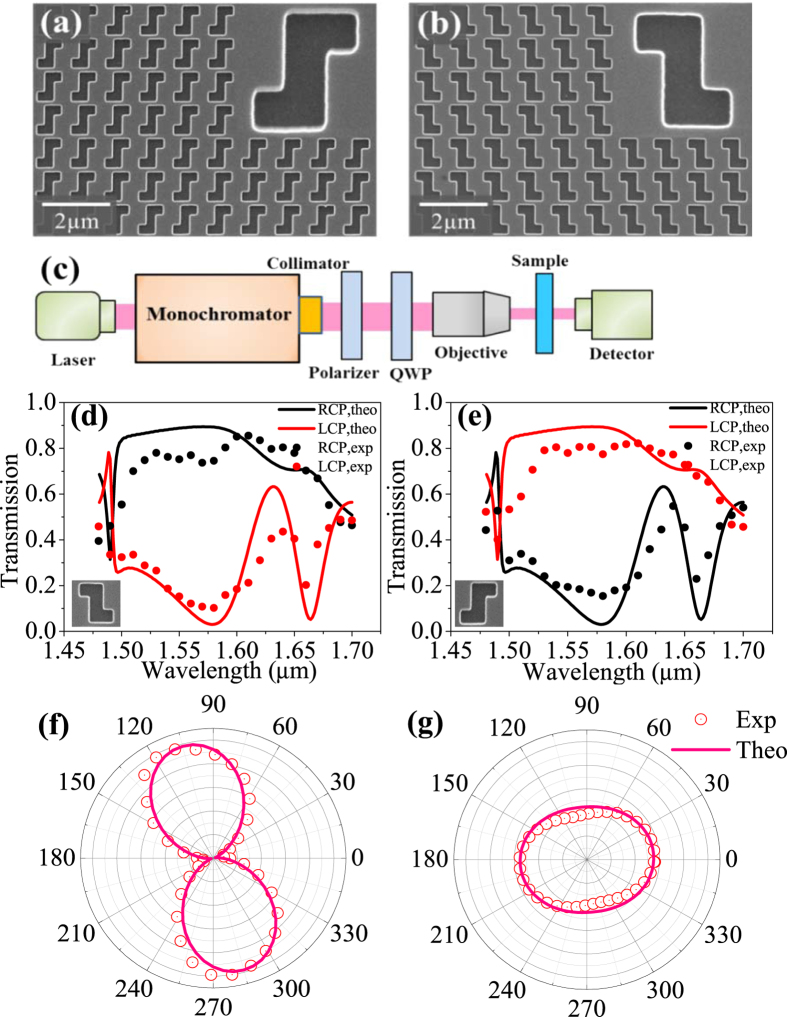
Transmission spectra and chiral metasurface. Scanning electron micrographs of the fabricated left and right-handed chiral metasurface structures (**a**) left-handed; (**b**) right-handed. The insets show a unit cell of the chiral metasurface. (**c**) Schematic of a spectral CPD measurement setup; (**d**) Experimental results of the measured transmission of left-handed and right-handed circular polarization incident on the left-handed structure; (**e**) Experimental results of the measured transmission of left-handed and right-handed circular polarization incident on the right-handed structure. (**f**) Experimental polar diagrams for the polarization state of the transmission with RCP incident light at wavelength 1.56 μm. (**g**) Experimental polar diagrams for the polarization state of the reflection with LCP incident light at wavelength 1.56 μm.

Experimental verifications of the proposed all-dielectric 2D chiral metasurface are performed with both left and right-handed chiral metasurface arrays of silicon film, Fig. 5(a) and (b), on silicon dioxide substrate. Figure 5(a) and (b) shows the scanning electron micrographs (SEM) of top-view images of the fabricated left and right-handed chiral metasurface structures, respectively. The inset shows a unit cell of the chiral metasurface. From the SEMs, the dimensions of the fabricated all-dielectric 2D chiral metasurface are measured as P = 0.986 μm, L_1_ = 0.237 μm, L_2_ = 0.491 μm and W = 0.319 μm, which are very close to the designed parameters as that in Fig. 2(e) (blue line) when H = 250 nm. A detailed theoretical prediction of the performance based on the fabricated dimensions of the device is given in Supporting information.

Figure 5(d) and (e) show the measured transmission spectra with two different circularly polarized incident lights (i.e., left-handed and right-handed circular polarization) on the fabricated left-handed and right-handed chiral metasurfaces. Theoretical transmissions of the two different circularly polarized incidences are also shown in Fig. 5(d) and (e) for comparison. It is clear that a large transmittance dichroism between left-handed and right-handed circular polarization appears in the wavelength range from 1.53 μm to 1.62 μm, which is slightly red-shifted compared with the theoretical prediction. The discrepancies between the simulated and measured results can be attributed to the fabrication tolerance. In addition, the small difference of materials permittivity between that used in the simulations and that in experiments may contribute to the discrepancies as well. In the measurement, the objective also induces an oblique incident effect in the chiral metasurface.

The detailed polarization state of the transmitted and reflected light when a RCP and LCP polarized light is incident on the left-handed chiral metasurface is also measured and analyzed. Figure 5(f) gives the experimental polar diagram of the polarization state of the transmission with a RCP incident light at wavelength 1.56 μm, which was measured with another polarizer inserted between the sample and the detector. It is shown that the transmitted light is almost linearly polarized at the resonant wavelength 1.56 μm and the polarization azimuthal angle is close to vertical direction, which is in excellent agreement with the theoretically predicted in Fig. S2(a). Figure 5(g) shows the polarization state of the reflected light when a LCP light is incident on the chiral metasurface. It can be seen that the reflected light is an elliptically polarized light (close to circular polarization) at the wavelength of 1.56 μm, as predicted in Fig. S2(b). The transmitted and reflected lights slightly deviate from an ideal linear polarization or a circular polarization, respectively, as expected theoretically, considering the fact that the fabricated chiral metasurface structure slightly deviates from the precise dimensions in design and also the measuring error. More experimental results corresponding to a linearly polarized incidence are given in Supporting information.

## Discussion

In summary, we have proposed and experimentally demonstrated a high efficient circularly polarizing dichroism waveplate (CPDW) using CMOS-compatible all-dielectric chiral metasurface structure. Polarizing behaviors of the proposed structure are analyzed and examined both theoretically and experimentally, in which the structure functions as a transmissive quarter waveplate for one of either left- or right-handed circularly polarized incident light and a reflective mirror for the other at the designed wavelength. Further examinations show that a linearly polarized transmission light with invariable polarizing azimuthal angle and a circularly polarized reflection light can be simultaneously obtained for a linearly polarized incident light with arbitrary polarizing azimuthal angle. These unique functions along with the simple all-dielectric structure make the device distinctive from all the previously reported ones. The demonstrated ultracompact device could be useful inquantum measurements, quantum telecommunications, remote sensing and polarizing imaging with a highly integrated photonic platform.

## Methods

### Simulations

The performance of the circular polarized dichroism of the proposed structure can be characterized using finite difference time domain method (FDTD) (Lumerical FDTD solutions, Canada). The perfectly matched layers (PMLs) along Z direction and the periodic boundary conditions along X and Y directions owing to the periodicity of the chiral structures are assumed in the simulation. The dielectric properties of Si given by Palik are adopted^28^. Two types of circularly polarized light, i.e., left-handed circular polarization (LCP) and right-handed circular polarization (RCP), are assumed to be incident from the substrate side along Z direction, and the transmission of the circularly polarized incident lights are calculated.

### Sample fabrication

A 250 nm silicon film is grown on a silicon dioxide with PECVD technology. Only the top silicon layer is etched into the designed Z-shaped slot arrays. The nano patterns were first made with an electron beam lithography system using PMMA resist (Pionner@Entire 3, Raith). After post baking and development, inductively coupled plasma etching was used to obtain the final nanostructures and it ended with an additional 10% over etch to ensure that Si was removed completely in the patterned regions.

### Optical measurements

The optical transmission behaviors of the fabricated all-dielectric 2D chiral metasurface were measured at normal incidence with both rightand left circularly polarized light in the wavelength range between 1.45 μm to 1.70 μm using a supercontinuum laser source (Fianium, SC450) and an optical spectral analyzer (Thorlabs, PAX5710IR3-T). The broadband light was firstly directed to a monochromator and a collimator. A linear polarizer and a tunable quarter-wave plate (Alphalas GmbH, PO-TWP-L4-12-UVIR) were then used to generate the desired circular polarization light. The quality of the generated circular polarization was checked with an additional polarizer. The light was then focused onto the sample by an objective. The polarization state of the transmitted light was measured with an additional polarizer put after the sample. The polarization state of the reflected light was measured with a reflective optical system.

## Additional Information

**How to cite this article**: Hu, J. *et al*. All-dielectric metasurface circular dichroism waveplate. *Sci. Rep.* **7**, 41893; doi: 10.1038/srep41893 (2017).

**Publisher's note:** Springer Nature remains neutral with regard to jurisdictional claims in published maps and institutional affiliations.

## Supplementary Material

Supplementary Movie S1

Supplementary Movie S2

Supporting Information
