# Supplementary material for: All-dielectric metasurface circular dichroism waveplate
Source: Sci Rep. 2017 Jan 31;7:41893. doi: 10.1038/srep41893 (PMC5282559; doi:10.1038/srep41893)
Supplement: Supporting Information [file srep41893-s3.doc]

**All-dielectric metasurface circular dichroism waveplate**

**Jingpei Hu^1,2^, Xiaonan Zhao^1,2^, Yu Lin^1,2^, Aijiao Zhu^1,2^, Xiaojun Zhu^3^, Peiji Guo^1,2^ , Bing Cao^1,2*^and Chinhua Wang^1,2*^**

^1^College of Physics, Optoelectronics and Energy & Collaborative Innovation Center of Suzhou Nano Science and Technology, Soochow University, Suzhou 215006, China. ^2^Key Lab of Advanced Optical Manufacturing Technologies of Jiangsu Province & Key Lab of Modern Optical Technologies of Education Ministry of China, Soochow University, Suzhou 215006, China. ^3^School of Electronics and Information, Nantong University, Nantong 226019, Jiangsu, China.

*Corresponding author: E-mail: [bcao2006@163.com](mailto:bcao2006@163.com), *E-mail: [chinhua.wang@suda.edu.cn](mailto:chinhua.wang@suda.edu.cn)

**S1:Transmission and reflection of RCP and LCP incidences on the proposed chiral structure**

Figure S1(a) and S1(b) show the transmission, reflection and absorption of a RCP and LCP incidences, respectively, on a left-handed chiral structure with optimized parameters, P=0.98µm, L_1_=0.22µm, L_2_=0.50µm, W=0.32µm and H=0.215µm. It is seen that the transmission and the reflection spectrum show a well expected symmetry under both RCP (Figure S1(a)) and LCP (Figure S1(b)) incidences, and the optical absorption across the all-dielectric metasurface structure is almost zero in the operating wavelength band, which means that the incidence light with either RCP or LCP is either transmitted or reflected by the 2D chiral metasurface without any absorption inside the structure.

**Figure S1.** The simulated spectrum (transmission, reflection and absorption) of different circularly polarized incidences on a left-handed all-dielectric chiral structure. (a) RCP incidence and (b) LCP incidence.

**S2: Theoretical prediction of the performance based on the fabricated dimensions**

Figure S2(a) and S2(b) show the detailed theoretical analysis regarding the transformation of the polarization state of a RCP and LCP incidences with dimensions around practically measured: P=0.98µm, L_1_=0.24µm, L_2_=0.50µm, W=0.32µm and H=0.25µm. Figure S2(a) shows the RCP and LCP components in transmission when a RCP light is incident onto the left-handed chiral metasurface. As can be seen, both RCP and LCP components exist in the transmission when a RCP light is incident onto the left-handed metasurface. The overall polarization state of the transmission is a vector summation of the co-existed RCP and LCP components in the transmission. As a result, a linear polarization can be obtained at the wavelength of ~1.56µm, where the intensity of RCP and LCP components in transmission are equal. The ellipticity and azimuth angle of the transmitted light with RCP incidence at 1.56µm is 0.1 and 86.7°, respectively, compared to η=0 and θ=90^o^ of a linear polarization theoretically. Figure S2(b) shows the RCP and LCP components in reflection when a LCP light is incident onto the left-handed chiral metasurface. As expected, RCP component dominates in the reflection while LCP component in the reflection is nearly zero (I_LCP_ ≈ 0) at the waveband of 1.50µm -1.56µm in the case of a LCP incidence. This results in a circular polarized light in the reflection with ellipticity being 43.3 at 1.56µm, compared to η=45 of a circular polarization theoretically.

Figure S2(c) and S2(d) shows the ellipticity and azimuth angle of the transmitted and reflected light, respectively, when a linearly polarized light at wavelength of 1.56µm with different polarization azimuth angles (with respect to X-axis) is incident onto the designed chiral metasurface. As discussed in Eq. (1) in the article, it is seen from Figure S2(c) that a linearly or near-linearly polarized light can be obtained (originates from the RCP component after decomposition of a linear incident polarization) in the transmission with an ellipticity close to zero and a fixed azimuth angle (θ) at ~90° no matter what the polarization azimuth angle of the incident light is. Similarly, in Figure S2(d), it is seen that an elliptically polarized light can be obtained in the reflection from a linearly polarized incidence (originates from the LCP component of the linearly polarized incidence). It should be noted that the ellipticity of both transmitted and reflected light in the case of linearly polarized incidence is different from that of transmitted and reflected one in the case of purely RCP or LCP incidence because of the superimpose effect in both transmission and reflection caused by co-existing RCP and LCP components in the linearly polarized incidence.

**Figure S2.** (a) RCP and LCP components spectra in transmission when a RCP incident light transmits through a left-handed metasurface. (b) RCP and LCP components spectra in reflection when a LCP incident light is reflected from the left-handed metasurface. (c) Polarization ellipticity (η) and azimuth angle (θ) of the transmitted light with a linearly polarized incident light at wavelength 1.56µm; (d) Polarization ellipticity (η) and azimuth angle (θ) of the reflected light with a linearly polarized incident light at wavelength 1.56µm. Parameters are P=0.98µm, L_1_=0.24µm, L_2_=0.50µm W=0.32µm and H=0.25µm.

**S3: Experimental results corresponding to a linearly polarized incidence**

Figure S3(a) and S3(b) show the experimental polar diagrams for the polarization states of the transmission of a linear polarization incident light with azimuth angles 0º and 90º, respectively, at wavelength 1.56μm. It is shown that both the transmitted light in Figure S3(a) and S3(b) are nearly linear polarized at the resonant wavelength at 1.56μm and both the polarization azimuthal angles are close to vertical direction no matter what the azimuthal angle of the incident light is, which is in excellent agreement with the theoretically prediction in Figure S2(c). Figure S3(c) and (d) show the polar diagrams for the polarization states of the reflection of the linear polarization incident light with azimuth angles of 0º and 90º, respectively, at wavelength 1.56μm. It can be seen that the reflected light is an elliptically polarized light at the wavelength of 1.56μm, as predicted in Figure S2(d). As can be seen all the figures, the polarization states of the transmitted and reflected lights are in excellent agreement with the theoretical prediction.

**Figure S3.** (a) and (b) Experimental polar diagrams for the polarization states of the transmission of a linear polarization incident light with azimuth angle of 0º and 90º, respectively, at wavelength 1.56μm; (c) and (d) Experimental polar diagrams for the polarization states of the reflection of the linear polarization incident light with azimuth angle of 0ºand 90º, respectively, at wavelength 1.56μm.

**S4: Evolution movies of transmission and reflection of different circular polarization incidence on the left-handed chiral metasurface**

The transmission evolution of a RCP incident light on the left-handed chiral metasurface is shown in Movie S1with FDTD simulations, (avi).

In comparison, the reflection evolution of a LCP incident light on the left-hand chiral metasurface is shown in Movie S2 with FDTD simulations, (avi).
